# Understanding for whom, how and why Sydney Local Health District’s Integrated Response was Effective in Addressing COVID-19: A Critical Realist Qualitative Study

**DOI:** 10.5334/ijic.5991

**Published:** 2022-02-11

**Authors:** Hueiming Liu, Darith Liu, Corey Moore, Lisa Parcsi, Ferdinand Mukumbang, Denise De Souza, Miranda Shaw, Lou-Anne Blunden, Teresa Anderson, John Eastwood

**Affiliations:** 1 Sydney Local Health District, NSW Health, AU; 2 Sydney Institute for Women, Children and their Families, AU; 3 The George Institute for Global Health, University of New South Wales, AU; 4 Department of Global Health, Schools of Medicine and Public Health, University of Washington, Seattle, WA, Washington, AU; 5 Ingham Institute for Applied Medical Research, Liverpool, NSW, AU; 6 Torrens University, AU; 7 School of Public Health and Community Medicine, University of New South Wales, AU; 8 Menzies Centre for Health Policy, The University of Sydney, AU

**Keywords:** COVID-19 pandemic, qualitative research, integrated care

## Abstract

**Introduction:**

Australia has been comparatively effective in preventing the transmission of COVID-19. The Sydney Local Health District [SLHD] used a “whole of health” integrated approach to respond to the pandemic. The aim of this study was to understand for whom, how and why this response worked, to inform a sustainable system transformation.

**Methods:**

A critical realist qualitative study was conducted with 20 purposively selected key informants. Data were collected through in-depth interviews and analysed using thematic analysis guided by abduction and retroduction. The five strategies of the WHO integrated people-centred health services framework was used to guide the overall study.

**Results:**

An enabling environment of a strong governance, emergency preparedness, a committed and adaptable workforce, and a strong core infrastructure underpinned SLHD’s effective response. With a culture of embracing innovation, the district adapted virtual care to effectively quarantine people through their special health accommodation, and coordinate care across tertiary and community services. The established interagency relationships prior to the pandemic, enabled service directors to quickly integrate their services, which empowered and engaged the community [and staff], working across relevant sectors to provide care “where the people are”; reaching marginalised populations, and reducing community transmission.

**Discussion and Conclusion:**

The SLHD’s progress towards a ‘whole of health’ approach, empowered and enabled the district to effectively work within and across sectors to address the pandemic in a people-centred manner. Sustaining the contextual conditions and mechanisms, that facilitated effective integration, will be beneficial beyond the pandemic.

## Introduction

Several countries with mature health systems have struggled to absorb the shock of the coronavirus disease of 2019 [COVID-19] [1,2,3,4], resulting in worsening health inequities due to system fragmentation. The challenges posed by the COVID-19 pandemic have accentuated the need to develop and embrace integrated health and social service systems [5].

As of March 4 2021, globally about 115 million people were infected with COVID-19 and 2,567,404 deaths were recorded. With 29,007 cases and 909 deaths, Australia has been relatively effective in preventing and managing the COVID-19 pandemic compared to other countries [6]. In addressing the pandemic, the Australian Government’s aims included to: “*minimise the number of people becoming infected or sick with COVID-19, minimise how sick people become and the mortality rate, manage the demand on our health systems, help [people] to manage [their] own risk and the risk to [their] family and community, support work towards a vaccine, make a future vaccine available to Australians for free*.” [7]

The state of New South Wales in Australia has 17 local health districts. The Sydney Local Health District [SLHD] and South Eastern Sydney Local Health District, being close to the Sydney international airport, were jointly in charge of quarantining returning travellers. Local health districts have a statutory responsibility for the provision of State funded health services within a defined geographical area. Given that international travellers are the main source of internationally imported transmissions, having a robust screening and quarantine system is critical. Unpacking how SLHD was effective in minimising the number of people being infected and becoming sick, may provide transferable lessons, and inform the sustainability of a resilient health system.

SLHD activated their emergency pandemic plan in February 2020 and adopted a “whole of health” approach in their response. The NSW “whole of health” programme transitioned from an earlier “whole of hospital” approach that was focused on hospital avoidance and post-discharge programmes. The “whole of health” approach has an expanded the initial service partnerships to include, for example, SLHD significant primary health care workforce within service areas such as mental health, community health, sexual health, child and youth health, and aged care. Those services are strongly engaged with communities and partner agencies. The primary health care response of SLHD included community screening clinics, and wellbeing clinics in partnership with NGOs and interagency partners.

We summarised this integrated response using documentary analysis of key situational reports. The district’s integrated response incorporated the different agencies and teams’ roles across functions of 1] rapid screening and testing; 2] reaching the community; 3] effective quarantine and ongoing care; and 4] infrastructure, pathology and staff education [8]. The in-depth description of our response, in the first 6 months, has been written up as a case study [8]. It showed how SLHD had to quickly ‘absorb’ the exponential requirements for screening, surveillance, and tracing, and also ‘adapt’ their practices to better reach the community and effectively quarantine, and also ‘transform’ services to cater for high-risk marginalised populations. Following a first wave community outbreak in early 2020 COVID-19 was eliminated from community spread until June 2021. We have previously reported in an audit of the SLHD public health unit management of the first wave of COVID-19 [9]. Based on NSW health reports, as of 3 March 2021, NSW health district conducted 382,359 COVID-19 tests, with an average of 47/1,000. Overall, SLHD was effective in conducting surveillance and screening and tracing. With a test rate of 53/1,000, there were no new cases, or cases with unknown source [10]. The COVID-19 pandemic response presented SLHD’s decision makers with a unique opportunity to reflect upon their health systems’ resilience — ‘ability to prepare for, manage [absorb, adapt and transform] and learn from shocks’ [11].

In this paper, we sought to unearth **how, why and for whom the SLHD integrated COVID-19 response worked**, to inform sustainable system transformation. Findings from this study can help maintain the SLHD gains that were catalysed through the pandemic’s response towards a people-centred health system, connecting the healthcare system with other human service systems [e.g. Housing, vocational] to improve service and clinical outcomes. Evaluating their COVID-19 response will help to examine the relevance of integrated people-centred services, a commitment by global governments to advance universal health coverage [12]. We hope that findings will also elicit transferable lessons globally.

### Ethical approval

Ethical approval was obtained from the Human Research Ethics Committee [Royal Prince Alfred Hospital Zone] of the SLHD, X20-0310.

## Methodology and methods

### Research paradigm

Our study was underpinned by the Critical realist ontological position, which proposes that ‘what actually exists i.e., reality’ is stratified and independent of empirical outcomes and human knowledge of the world, and therefore all knowledge is ‘theory laden’. Epistemologically, Critical realism submits that social structures can trigger causal mechanisms leading to an observable phenomenon [13]. A Critical realist methodology enables the researcher to uncover the “hidden” causal mechanisms that are postulated to be contributing to the empirically observed phenomenon. Critical realism also examines the contribution of historical and current context within which integrated health and social care interventions are operating [14].

In this study, the outcomes are the low COVID-19 transmission and mortality rates, and the observable phenomenon is the integrated SLHD response. We theorised that the ‘unseen’ causal mechanisms are embedded in the 5 strategies of the WHO integrated people-centred health services framework of: 1] empowerment and engagement of communities, 2] coordination of services, 3] reorientating the model of care towards primary health care [as defined by WHO as a whole of societal approach to health across the continuum from promotion to pallative care, to be as close to the people], 4] good governance and accountability, and 5] creating an enabling environment; and that the actions are contingent on the context of SLHD healthcare institutional structures [12]. We sought to better understand the important contextual conditions that activated [or did not activate] these causal mechanisms to achieve an integrated response that was effective in reducing the spread of the COVID-19 pandemic in SLHD.

### Sampling

Using purposive sampling, we mapped the key informants of the COVID-19 integrated response based on the findings of the documentary analyses done for the descriptive case study [8]. Identified key informants included SLHD managerial staff of public health unit, integrated care and population health, Royal Prince Alfred Virtual Hospital, and community health. These key informants were sent an email to participate in the interviews. We also asked interviewees for suggestions of other key informants that may provide a different perspective [snowballing sampling]; and two other key informants were suggested. Of 26 invitations sent, 20 accepted; and of those that did not accept, reasons cited were mainly around the lack of time to commit to the study. There was representation across all agencies among the participants who agreed to participate.

### Conduct of interviews

Key informants responsible for the decisions of the district’s response to the COVID-19 pandemic during Feb 2020 to Nov 2020 were interviewed. There were thirteen female and seven male interviewees, who were a mix of senior management staff and clinical directors. These semi-structured interviews, lasting between 30–60 minutes were conducted by DL, CM and HL via videoconference or face-to-face [while observing all social distancing protocols] between July to Nov 2020. Verbal informed consent was obtained. The interview guide was developed based on initial observations by authors, of improved integration across agencies to address the COVID-19 pandemic, and we sought to better understand the scope and extent of integration, the context, and the mechanisms as to how it happened. This included broad questions relating to each agency’s roles, responsibilities, and decision-making processes in the planning for and carrying out the COVID-19 pandemic response. Overall, we asked questions relating to how the core services were integrated, and what and how aspects of the integration worked or not. Additional probing questions across the domains, were added as we iteratively analysed our interviews to inform our line of inquiry. [See interview guide, Supporting Table 1].

### Analysis

The interviews were analysed by HL and CM who are public health advanced trainees and DL, who is a medical administration registrar. The interviews were audio recorded and transcribed verbatim by a professional company. Each transcript was checked either by DL or HL against the original audio file for accuracy. Data were managed using NVivo version 12. HL, CM and DL had in-depth discussions after each interview to synthesis key points from each interview, and triangulate emerging findings across the different perspectives. After adjusting the codebook following a discursive process, we shared the remaining transcripts and coded all transcripts. HL reviewed the coding, and further summarised codes with similar meaning, and streamlined the codebook [Supporting Table 2]. Constant comparison across participants’ transcripts were done for the major nodes [15], to derive the themes through abduction and retroduction informed by the context-mechanism-outcome heuristic tool.

We used deductive and inductive analyses informed by the UK Medical Research Council guidance on complex interventions [16,17] to uncover major nodes of context, intervention description, mechanisms, implementation and outcomes [12]. Abductive and retroductive thinking were applied to unearth the causal mechanisms acting within the WHO’s five strategies and to hypothesise how they generated the observed outcomes. First, we connected the context constructs to relevant generative mechanism[s] identified to postulate the outcome [18]. To this end, based on the data analysis and retroductive theorising process, we made aligning to the five WHO strategies.

## Results

We have organised our findings into key themes as seen in ***Table 1***, which summarises the linked intervention modalities [input] to the relevant context conditions, and mechanisms to the observed outcomes. These explanations are presented as propositions which are theories about the conditions and causal mechanisms that need to exist to explain the observed outcomes. As argued by Bhaskar they are temporarily completed analyses that are open to future “contestations and corrections” [19].

**Table 1:** Logic model of SLHD integrated response to summarise our findings.


CONTEXTUAL PROBLEMS	INTERVENTION/RESPONSE [INPUT]. DESCRIPTION OF SLHD INTEGRATED RESPONSE WHICH ADDRESS THE CONTEXTUAL PROBLEMS, ACROSS THE PHASES OF ABSORB, ADAPT, AND TRANSFORM FOR HEALTH SYSTEM RESILIENCE	ACTIVATION OF THE THEORISED WHO 5 STRATEGIES AS MECHANISMS WITHIN LOCAL CONTEXTUAL SYDNEY LOCAL HEALTH DISTRICT STRUCTURES TO RESULT IN PROXIMAL OUTCOMES	AUSTRALIAN GOVERNMENT HEALTH POLICY OUTCOMES FOR THE COVID- 19 RESPONSE

Global pandemic timelines that required a rapid response to address the uncertainties, and to prevent overwhelming of the health system, and address staff and communities fears.	Integrated response to ABSORB: Rapid public health response needed such as the flying squad, guidelines for infection control, care of positive patients, preparing the system, quarantine measures to be put in place	The context of strong leadership, established infrastructure, and epidemic preparedness, activated mechanisms of accountability and governance [4], to result in swift responses and early detection of COVID-19. The context of underlying partnerships and a culture of innovation, activated mechanisms of coordination [2] within and across agencies, to result in efficient and streamlined care.	“Minimise the number of people [including staff] who become infected or sick with COVID19; Minimise how sick these people become & how many people die; Reduce the burden on our health systems, to continue to provide the regular health care ; Help Australians to reduce their own risk and the risk to their families and communities; Delivery of Vaccine”

Overseas experience [e.g., UK], highlighted how hospitals were overwhelmed, and the need to address the needs of the community and reach the vulnerable populations	Integrated response to ADAPT: Models of care to expand the reach into the community, facilitated by the telehealth/virtual hospital that was being piloted, community well-being clinics, drive through clinics set up, aged care outreach teams empowered. Engaging other sectors to address the marginalised populations.	The context of SLHD’s vision to keep all community and staff safe activated the mechanisms of empowering and engaging diverse communities [1], resulted in reduced community transmission and anxiety. The context of inter-sectoral partnerships and virtual care brought services to ‘where the people are’ activated mechanisms of reorientating the model of care [3], resulted in more equitable access to screening and testing.	

In face of the uncertainties of subsequent waves, there is a need for sustainability of the integration of the model of care, workforce deployment.	Integrated response to TRANSFORM: strong infrastructure built quickly, and measures to sustain this while providing usual care in a more efficient manner.	The context of SLHD underlying ‘whole of health’ approach, and accountability structures activated mechanisms of an enabling environment [5] for the pandemic response that resulted in transformational change.	


*Note*: WHO Framework causal mechanism legend – 1] empowerment and engagement of communities; 2] coordination of services within and across agencies; 3] reorientating model toward primary health care; 4] governance and accountability; and 5] enabling environments.

Additional illustrative quotes across the themes are in ***Table 2***.

**Table 2:** Illustrative quotes across the themes.


THEME	ILLUSTRATIVE QUOTES

**The context of strong leadership, established infrastructure, and epidemic preparedness, activated mechanisms of accountability and governance, to result in swift responses and early detection of COVID-19**.	*“We have a strong Chief Executive who took a strong lead very early and worked very strictly with the three C’s, Command, Control and Communication. So, there was no doubt in which way we were moving forward, there were Action Plans developed, meetings held, …*” [Participant 14]

**The context of underlying partnerships and a culture of innovation, activated mechanisms of coordination within and across agencies, to result in efficient and streamlined care**.	“*a Virtual Care model has a role in a pandemic response and can surge very quickly. Has very different infrastructure requirements to a traditional hospital. For example, we have 350 m^2^ here. We’ve been able to treat 3,500 patients… So, it’s a much more efficient and leaner model*” [Participant 9]. “*the chief executive is also pretty tech savvy to an extent, you know, she is very …a very innovative thinker, so if we present to her an idea that solves a sticky problem, she’ll have, you know, a couple of questions but then she’ll back you a thousand percent and, you know, give us the money that we need to pull that off*” [Participant 11].

**The context of SLHD’s vision to keep all community and staff safe activated the mechanisms of empowering and engaging diverse communities, resulted in reduced community transmission and anxiety**.	“*people have universally, across the district, accepted that life is different and that their roles have changed and that they’re willing – the willingness, I think, has just been unbelievable, for people to shift and pivot and change focus and do extra things, or do different things, or, you know, put their hand in and say, Yep, I’m willing to step up*” [Participant 18]. *“People were really frightened. A lot of those residents. And so, they were able to meet with social workers and other mental health people to actually address their issues. Many people needed assistance. They needed meals. They needed other things… So, the translation of that amazing amount of work, amazing amount of material, has been a really important – and we’ve worked very closely with local councils to try and get ideas off them and how we can assist with different strategies, with different groups*” [Participant 16].

**The context of inter-sectoral partnerships and virtual care brought services to ‘where the people are’ activated mechanisms of reorientating the model of care, resulted in more equitable access to screening and testing**.	“*We set up wellbeing clinics in the community, in locations that were easily walkable for primarily, people sleeping rough and people in boarding houses…. We got a heat map where people from the homelessness count and where boarding houses are. And we mapped where we could reasonably provide these wellbeing clinics….*” [Participant 13] “*if the [aged care] facilities are virtually enabled, then there’ll be means of enabling video conference, communication with families and others in the event of a problem with lockdown to overcome … or to lessen the … the severe effects of isolation*” [Participant 10]

**The context of SLHD underlying ‘whole of health’ approach, and accountability structures activated mechanisms of an enabling environment for the pandemic response that resulted in transformational change**.	“*For example, the Waterloo pop-up, you know, where we started to understand about the Public Health issues that were emerging from Melbourne … the Public Housing issues… and we had to learn from that… So that’s happening a lot more than it ever did*” [Participant 7]. “*Very little has been shifted to the Community sector over the years. And that’s even in terms of building infrastructure. Whenever you hear about infrastructure, it’s always acute hospital infrastructure…. But it certainly has brought everyone a lot closer together, which is a terrific thing across the district, rather than seeing, you know, “theirs” and “ours” or whatever. I think there’s more respect there, for what everybody does*” [Participant 16].


### Accountability and Governance

***Proposition: The context of strong leadership, established infrastructure, and epidemic preparedness, activated mechanisms of accountability and governance, to result in swift responses and early detection of COVID-19***

Many informants described how the existing NSW Health and the health district governance structures facilitated the adaptation to the ‘Incident command’ strategy as per their underlying pandemic and disaster management plan structure in place for SLHD. This meant that staff with extensive disaster management experience were put in charge to execute previously established [and practiced] plans, and the Chief Executive became the ‘*commander, oversight person*’ who was strongly supported by a network of clinicians and managerial staff. The SLHD executive team described foreseeing the pandemic in February 2020, which was prior to WHO’s declaration. Emergency meetings were then held to establish clinical pathways and management protocols. This included the need to rapidly increase existing infrastructure such as number of Intensive care beds, when it was apparent that mature health systems globally [e.g., Italy] were being overwhelmed, and experiencing limited supplies of COVID-19 tests and personal protective equipment. Many participants described how sharing a common vision to “*make sure we meet all of our obligations, keep the community safe, and keep our staff safe*” [Participant 19] was crucial. This vision grounded their roles and responsibilities despite great uncertainty and evolving international advice including case definitions, testing criteria, and public health orders.

All the interviewees highlighted that strong leadership resulted in a quick and rapid response to absorb the shock of the pandemic, to be able to implement action plans, and sustainably manage the ongoing testing, screening, and quarantine protocols. Moreover, the underlying SLHD governance structure in accordance with New South Wales state structures, meant the district “*had all the systems and structures, so we could just move it in*” [Participant 19] through a ‘*hub and spokes*’ [Participant 14] model of operation. This included for instance, setting up onsite screening clinics promptly within two hours once a localised outbreak was recognised, screening at domestic and international airports, train stations; and as the testing requirements grew, deploying relevant surge workforce from other departments to assist in pandemic response.

### Coordination within and across agencies

***Proposition: The context of underlying partnerships and a culture of innovation, activated mechanisms of coordination within and across agencies, to result in efficient and streamlined care***

Addressing the pandemic required significant coordination and communication across the whole district, including infection control [e.g., use of personal protective equipment, consistent signage in facilities], information technology and streamlining electronic health records across systems. Participants echoed how this resulted in closer communication with others in the district, breaking down siloes of communication and strengthened relationships between managerial staff. Many participants across the departments [e.g., pathology, public health, community health] described having others on ‘*speed dial*,’ regardless of the hierarchical structure. This allowed for work to be actioned rapidly. Replacing face-to-face meetings with video conferencing also enabled communication and allowed senior clinicians and directors from other facilities to meet regularly with minimal impact on their clinical load.

SLHD had an underlying culture of innovation prior to the pandemic. The executives highlighted how newly established virtual care at the Royal Prince Alfred [RPA] hospital [RPAvirtual] could immediately be pivoted to supporting the special health accommodation to quarantine COVID-19 positive patients and returning travellers. Senior clinical and managerial staff had to quickly “*design and implement a remote monitoring programme for COVID community patients at home*”, which resulted “*in a very close rapid relationship between the Public Health Unit and a clinical service, which is highly unusual and that that was a real cornerstone of the response within our District*.” [Participant 1]. Managerial staff of the RPAvirtual hospital described how the pandemic enhanced it being a viable model of care, which could lead to system efficiencies.

RPAvirtual working closely with the SLHD Special Health Accommodation, to provide effective quarantine. The SLHD special health accommodation grew from an initial “*inhouse accommodation for the district*” that was available prior to the pandemic, for patients’ families who travel from outside the district for care. The in-house accommodation provided a “*head start*” [participant 14] at the start of the pandemic, such that as the numbers to be quarantined grew, NSW Health tasked SLHD to “*hire out a number of hotels, which became [the] special health accommodation.*” This required rapid establishment of a system to be able to support over 500 patients rapidly [Participant 14].

A strong information communication technology [ICT] system was crucial to the integrated response. Their demand greatly increased with the transition of staff working from home, reliance on tele-health consultation, coordination for RPA virtual and special health accommodation. ICT staff described that they “*really started thinking more agile and thinking more creatively, … virtual huddles to collectively and collaboratively come up with solutions to the problems that the district was facing*” [Participant 11]. This included designing patient and provider-facing applications, and integrated information systems across care points. For example, necessary diagnostic and management systems were incorporated on a ‘gigantic trolley’ to swiftly set up COVID-19 testing in hot spots, within hours of notification. Staff described how executive leadership facilitated ICT staff to innovate and problem solve. Moving forward, the key ICT role played in system integration has led “to *a full district level digital health strategic plan… fully partnered with all of the departments across the district*” [Participant 11].

### Empowering and engaging diverse communities

***Proposition: The context of SLHD’s vision to keep all community and staff safe activated the mechanisms of empowering and engaging diverse communities, resulted in reduced community transmission and anxiety***

All the participants described a common vision of “*being in it together*’ to reduce community transmission and anxiety. Staff were therefore happy for workforce deployment to take place and to adapt to new roles. For example, experienced population health and dental health staff were deployed and trained to assist the Public Health Unit in contact tracing; highly experienced surgical and critical care nurses were trained to be the ‘Tiger team’, an elite and mobile squad who could rapidly screen and test for COVID-19 and educate and train additional staff in community settings.

Managers expressed how specialised expertise through population health units such as Diversity Hub, Health and Equity Research Unit helped engage and empower the diverse communities. For example, education materials were translated to different languages and distributed widely to reach the culturally and linguistic diverse communities. This reassured those communities regarding food insecurity and growing community anxiety. Inter-sectoral collaborations were initiated which set up community well-being clinics in geographical locations that were associated with lower socio-economic status, and food and care packages were distributed. Furthermore, education and flu vaccinations were offered at these clinics. Groceries were also delivered through the existing aged care outreach staff to elderly in their homes, which minimised their exposure in the community. To allay community concerns. A SLHD hotline was set up to take calls and to report on test results as soon as possible. Likewise, SLHD partnered with community members and local organisations such as Lebanese Muslim Association, local councils, Housing and Redfern Aboriginal Medical Service to address the communities’ needs in a culturally safe and consistent manner.

Underpinning the commitment to keep SLHD staff and their families safe, the higher-level executives said that the COVID response was “*a marathon and not a sprint*” and how “*watching overseas and seeing the impact of COVID-19 on staff and the community. It was about, “how do we react quickly and safely?” and making sure that we kept our health care workers safe. Because if you lose your health care workers, then the rest of your response is problematic… and by looking after the staff, it means that we’re going to be sustainable and get through it*…” [Participant 19].

This meant for example, apart from reducing community transmission, managers had to quickly learn on the go and be cognisant on the mental toll that the pandemic took on the staff and provide adequate leave arrangements and access to counselling services. One of the strategies employed by one of the managers to address the mental toll, was the importance to stop and reflect: “*we all talked about all the things that we had done to support the response from our very localised level. And it just made people feel really proud of the work that they had done*.” [Participant 18].

### Reorientating the model of care

***Proposition: The context of inter-sectoral partnerships and virtual care brought services to ‘where the people are’ activated mechanisms of reorientating the model of care, resulted in more equitable access to screening and testing***

Diverting patient management away from the hospital and delivering screening and patient services in the community ‘where the people are’ was a key strategy employed by SLHD executives. This included actively engaging marginalised people in the community through outreach using community-wellbeing clinics, working closely with the Aged Care facilities, establishing the first drive through clinic in Summer Hill, establishing mobile testing vans in marginalised communities, and caring for the community through RPAvirtual.

Importantly, interviewees highlighted how inter-agency partnerships had to work closely on those initiatives, to ensure vulnerable patients did not fall through the gaps. “*We’ve also been working with our partners more broadly, so in terms of integrated responses for example, with Out of Home Care and with Department of Communities and Justice, we actually set up a vulnerable client’s group, where there was education, the [Primary Health Network] PHN, [Department of Communities and Justice] DCJ, Out of Home Care, it’s led by our Child Protection Strategy Coordinator Director, where we actually identified all our vulnerable clients…to just ensure we’re picking up any people that might fall through the cracks at this time*” [Participant 7].

Population and community health senior staff were deployed strategically to deliver the community-based models of care. However, participants expressed concerns about ‘business as usual’ work being neglected and the impacts of this as ‘the marathon’ went on. For example, the backlog of elective surgical cases, the reduction in home visits for first time mums, and unintended consequences of public health orders in cases of disclosure from sexual assault victims where assault took place while breaking public health orders.

The health care and wellbeing of the older population was also highlighted by a few interviewees as a significant challenge, given the tragic COVID-19 outbreaks in aged care facilities associated with high mortality. A clinician described- “*you’re in a nursing home and you’re locked down and you’ve not been able to see your family for months, and you’re towards the end of your life. It’s pretty difficult. People become depressed and stop doing things and are never able to start doing. People are isolated at home lose their social networks. It’s been socially devastating and it’s not something the older population are benefitting from*” [Participant 10]. Virtual care supporting local staff in clinical assessments was proposed as a potential solution moving forward, and also allow aged care residents greater communication with families and “*lessen the severe effects of isolation*” [Participant 10].

### Enabling Environment

***Proposition: The context of SLHD underlying ‘whole of health’ approach, and accountability structures activated mechanisms of an enabling environment for the pandemic response that resulted in transformational change***

All the informants described how the pandemic brought the stakeholders together. Facilitators included accountability mechanisms of being data-driven, drawing on lessons learnt from other districts, and utilising the governance and financing structures. Several executives in community health described how the integrated response highlighted the value of the continuum of care [preventive and community health] as per the ‘whole of health’ approach. This is illustrated by the following quote: “*the importance to localise it, to place and people, you know, to actually have the analyses going on and analyse what we’re doing. And I think that’s the essence of Community Health is about localising all services to place and people. And I think everyone’s realised how important that is.*” [Participant 7] *SLHD* management described that they highly valued evaluation and research alongside service delivery, such as with RPAvirtual, and moving forward will use patient centred measures to understand the acceptability of the COVID-19 response for the community.

Several interviewees described the value of non-acute care agencies in the prevention of transmission, in limiting the potential surge in demand for critical care beds due to COVID-19. A participant stated that the integrated response provided “*a lot of visibility to the very hidden parts of the District that aren’t about patients and beds” and that there was a growing recognition that, “if we don’t get that contact right in the community, you’re never going to have enough Intensive Care Unit beds*” [Participant 4]. There was a hope moving forward to learn from how they integrated their care during the pandemic response and to continue the partnerships internally and externally so as to have a sustainable model of care.

Similarly, the success in the inter-sectoral approach in “*suddenly get [ting] virtually everybody off the street*” demonstrated that a “*housing first*” approach was feasible, and likely to have a sustainable impact. One of the directors described “*the Specialist Homelessness Providers are very active, and they would be lobbying DCJ [Department of Communities and Justice] to – so you know, DCJ have released [the] 36 million Together Home package, which is money that goes to Community Housing Provider for people who are rough sleepers to get them into community housing. Wrap around support… I mean, we’ve known that that’s what’s needed for as long as there have been homeless people. So, it would be interesting to see… how long will the funding continue*…” [Participant 13].

Importantly, the impact that RPAvirtual had in the response helped to overcome some clinicians’ initial reservations about virtual care: “*There was resistance from some…you know, cynicism about Virtual Care and what it could deliver… But, you know, if the pandemic hadn’t come along… We would have just rolled along with the Virtual Hospital Model of Care doing various bits and pieces, but that broad acceptance of Virtual Health as a viable alternative would have taken years*” [Participant 9].

Transformational system change seems to have resulted from the inclusive leadership and decision-making structures within the district and across the state. This included having regular meetings so that “*the Directors and, Staff Specialists will dial into that just to get, sort of, coordinated advice across New South Wales about the changes and, how to manage issues that come up, because obviously issues that come up here are not just localised to our district*” [Participant 6]. This contributed to knowledge exchange. For example, sharing how some of the ICT innovations could be adapted for use in facilities external to SLHD. An interviewee described “*We’re actually leading the state… As far as the COVID screening clinic pre-registration application, we’ve had pretty big talks with both eHealth and ministry and every other CIO all across the districts in New South Wales and everyone wants it*” [Participant 11]. Similarly, another interviewee described, “*we were very keen to share and be able to share our resources right across the State to anyone who needed them*” [Participant 18]. The streamlined collective response provided consistent information and made it less confusing for the public. “*We tried to make sure that the information we were consistently giving out was the New South Wales Health website…So, whilst it seems like there was lots of information around COVID right around the world, the information, the one source of truth for us, was always pointing back to that New South Wales Health website*.” [Participant 18].

Adequate resourcing for the response was enabled by underlying data-driven culture. The performance unit were able to quickly pull together the metrics and outcomes of the COVID-19 response activities, which justified the need for ongoing funding from NSW Health. In addition, there were strategic decisions about utilising an appropriate mix of federal and state financing structures to optimise procurement and provision of services according to need. Interviewees provided examples for this including pathology testing, availability of personal protective equipment and screening. For instance, SLHD was able to meet the exponential demand for testing due to the strong guidance provided by NSW pathology, and a private-public partnership with private laboratories.

## Discussion

We sought to unpack the context and the generative mechanisms triggered to explain how SLHD was resilient in being able to prepare for and manage the shock of the pandemic. An enabling environment of a strong governance, emergency preparedness, a committed and adaptable workforce, and a strong core infrastructure underpinned SLHD’s swift response. With a culture of embracing innovation, the district adapted virtual care to effectively quarantine people through their special health accommodation, and coordinate care across tertiary and community services. The established interagency relationships prior to the pandemic, enabled service directors to quickly integrate their services, so as to empower and engage the community [and staff], and work across other sectors to provide care “where the people are” reaching marginalised populations, and reducing community transmission. Thereby enabling SLHD to meet the Australian government’s aims of reducing the transmission of COVID, and number of people falling sick.

### Comparison to literature and implications

Our findings highlight how strong leadership and governance structures, together with epidemic preparedness, were essential. This is consistent with a global phenomenon of how effective leadership was key in the outcomes of countries such as Singapore, Taiwan and South Korea [20]. Indeed, experts have criticised how Global Health Security Index, an assessment of countries’ preparedness for pandemics, did not accurately predict the inverse outcomes in highly ranked countries in 2019, such as USA and UK [21]. The Global Health Security Index includes categories such as the ability to prevent, detect and report pathogens, compliance with international norms and the risk environment. Our findings align with recent suggestions that the next iteration of the Global Health Security Index should also include measures of effective governance and societal well-being; by reviewing countries’ decision-making mechanisms during the COVID-19 pandemic [21]. Apart from strong ‘top-down’ leadership, our findings highlight the importance of participatory and nimble leadership [22]. This includes the ability for leadership to listen to solutions suggested by front-line workers, and readily pivot existing care structures [e.g., RPAvirtual to assist in hotel quarantine], while ensuring usual health care continues. Such participatory leadership will be needed to ensure a more agile health system, as advocated for by health system researchers, to be able to respond swiftly through an iterative cycle of monitoring and learning when implementing strategies [23].

We found significant value in having the ‘whole of health’ approach with other sectors integrated alongside the public health response. Communities were empowered to adhere to public health advice that was consistently delivered, health services were streamlined, and sectors of housing, health, education worked closely to meet communities’ needs. Similar recommendations have been made by others, to have greater integration of health and economic response, for better societal outcomes [22,24,25]. Indeed, in a recent review of 28 countries’ COVID response using a health system resilience framework, it was highlighted the health sector had to draw on experiences from other sectors towards a ‘whole-of-government’ using a range of evidence-based research of policies [26].

Our findings highlight how important a data driven response is, in order to advocate for and sustain funding in the context of federal and state health finance structures, across sectors, and private-public partnerships [e.g., pathology, private health insurers]. In the context of limited resources, policy makers often have to choose between investing in global health security, and universal health coverage resulting often in fragmented health services [20,21,22]. In contrast, our findings indicate that the ‘whole of health’ approach enabled our effective response towards the pandemic. The increasing recognition and respect across agencies brought us together and accelerate the momentum towards providing screening, education ‘where the people are’ and prevented the scenario of overwhelmed hospitals and health staff falling sick [27]. Indeed, our findings support Loeweson’s recommendation that globally apart from implementing the security agenda through necessary measures such as public health orders, border closures and quarantine; participatory leadership and strong governance enabling service integration equitably and is rights-based is vital, to address increasing health inequalities exposed during this pandemic [22]. We highlighted the importance of maintaining the contextual conditions and mechanisms to strengthen a people-centred integrated health system, a recommendation also highlighted in the WHO COVID-19 strategic preparedness and response plan [28]. As Australia rolls out the vaccination nationally [29], it will be crucial for SLHD to maintain the social dynamics of close relationships between individual managers, agencies, sectors, with mutual respect across the tertiary and community health service agencies to better serve our community [30].

### Strengths and limitations of this study

Using the critical realist paradigm has been a useful approach in our qualitative study to unpack the decision-making process of the observed phenomenon of service integration. We confirmed the value of the WHO five strategies in our analysis, which will continue to guide our health district planning, investment, and service delivery. A limitation, however, was that while health system structures such as financing and legislation were alluded to during the interviews, greater information about how they impacted on the response, would require an in-depth policy analysis, which is beyond the scope of this study. Another limitation of our study is that our key informants and interviewers are all staff of SLHD, and responses to our proposed theories of what was working and not working may not have been entirely candid. However, comparing the informants’ responses across major nodes; triangulating the themes with the findings from the document analysis, and objective outcome indicators of SLHD activities- ensure the robustness and completeness of our analysis and findings.

## Conclusion

Our study demonstrates that SLHD using the ‘whole of health’ approach, was able to effectively absorb and adapt to the ‘shock’ of the pandemic. SLHD’s resilience as a health system provides transferable lessons to other health care systems, such as the need for strong participatory leadership and governance structures and epidemic preparedness; the value of “whole of health” integrated approach; and the need for data driven response to ensure equitable and efficient allocation of resources in a timely manner; and in doing be a learning system, that will transform to be sustainable and people-centred during COVID, and beyond.

## Additional File

The additional file for this article can be found as follows:

10.5334/ijic.5991.s1 Supporting Tables. Supporting Tables 1 and 2.

## References

[1] Oppenheim B, Gallivan M, Madhav NK, Brown N, Serhiyenko V, Wolfe ND, et al. Assessing global preparedness for the next pandemic: development and application of an Epidemic Preparedness Index. BMJ Glob Health. 2019; 4(1): e001157. DOI: 10.1136/bmjgh-2018-001157 PMC6352812 30775006

[2] Cole HV, Anguelovski I, Baró F, García-Lamarca M, Kotsila P, Pérez del Pulgar C, et al. The COVID-19 pandemic: power and privilege, gentrification, and urban environmental justice in the global north. Cities & Health. 2020; 1–5. DOI: 10.1080/23748834.2020.1785176

[3] Lal A, Erondu NA, Heymann DL, Gitahi G, Yates R. Fragmented health systems in COVID-19: rectifying the misalignment between global health security and universal health coverage. The Lancet; 2020. DOI: 10.1016/S0140-6736(20)32228-5 PMC7834479 33275906

[4] Sivashanker K, Duong T, Resnick A, Eappen S. Health care equity: From fragmentation to transformation. NEJM Catalyst Innovations in Care Delivery. 2020; 1(5).

[5] Stein KV, Goodwin N, Miller R. From crisis to coordination: challenges and opportunities for integrated care posed by the COVID-19 pandemic. Int J Integr Care. 2020; 20(3). DOI: 10.5334/ijic.5595 PMC7427683 32863805

[6] Coronavirus Resource Centre. Dashboard by the Center for Systems Science and Engineering at John Hopkins University [Available from: https://coronavirus.jhu.edu/map.html].

[7] Government response to the COVID-19 outbreak [Available from: https://www.health.gov.au/news/health-alerts/novel-coronavirus-2019-ncov-health-alert/government-response-to-the-covid-19-outbreak#:~:text=The%20Australian%20Government’s%20health%20response,demand%20on%20our%20health%20systems].

[8] Liu D, Liu HM, Moore C, et al. Sydney Local Health District’s Integrated Response to the COVID-19 Pandemic: A Descriptive Study (Submitted to International Journal for Integrated care, under review); 2021. 10.5334/ijic.5938 PMC9524298 36248070

[9] Jain N, Moore CB, Quinn E, Liu HM, Liu D, Heaton M, et al. Audit of the Sydney Local Health District Public Health Unit notification and contact tracing system during the first wave of COVID-19. Aust N Z J Public Health. 2021; 45(5): 526–30. DOI: 10.1111/1753-6405.13145 34473383 PMC8652577

[10] Health NSW. COVID-19 in NSW [Available from: https://www.health.nsw.gov.au/Infectious/covid-19/Pages/recent-case-updates.aspx].

[11] Thomas S, Sagan, A, Larkin J, Cylus J. Strengthening health systems resilience key concepts and strategies; 2020. 32716618

[12] WHO Framework on Integrated people-centred health services [Available from: https://www.who.int/servicedeliverysafety/areas/people-centred-care/en/.

[13] De Souza D. Educational change in Singapore and its ‘tinkering’ around the edges: A critical realist perspective. J Educ Change. 2018; 19. DOI: 10.1007/s10833-017-9314-z

[14] Eastwood JG, De Souza DE, Mukumbang FC. Realist Research, Design and Evaluation for Integrated Care Initiatives. Handbook Integrated Care. Springer; 2021. 629–56. DOI: 10.1007/978-3-030-69262-9_37

[15] Patton MQ. Qualitative research and evaluation methods. 3 ed: Sage publishers; 2002.

[16] Liu H, Lindley R, Alim M, Felix C, Gandhi DB, Verma SJ, et al. Protocol for process evaluation of a randomised controlled trial of family-led rehabilitation post stroke [ATTEND] in India. BMJ Open. 2016; 6(9): e012027. DOI: 10.1136/bmjopen-2016-012027 PMC5030603 27633636

[17] Liu H, Lindley R, Alim M, Felix C, Gandhi DB, Verma SJ, et al. Family-led rehabilitation in India [ATTEND]-Findings from the process evaluation of a randomized controlled trial. Int J Stroke. 2019; 14(1): 53–60. DOI: 10.1177/1747493018790076 30044209

[18] Mukumbang FC KE, Eastwood JE. Examining the Application of Retroductive Theorizing in Realist-informed Studies. International Journal of Qualitative Methods; 2021. DOI: 10.1177/16094069211053516

[19] Bhaskar R. Contexts of interdisciplinarity: Interdisciplinarity and climate change. In: Bhaskar R, Frank C, Hoyer K, Naess P, Parker J (eds.), Interdisciplinarity and Climate Change: Transforming knowledge and practice for our global future. London: Routledge; 2010. DOI: 10.4324/9780203855317

[20] Dalglish SL. COVID-19 gives the lie to global health expertise. Lancet. 2020; 395(10231): 1189. DOI: 10.1016/S0140-6736(20)30739-X PMC7194526 32222159

[21] Ravi SJ, Warmbrod KL, Mullen L, Meyer D, Cameron E, Bell J, et al. The value proposition of the Global Health Security Index. BMJ Glob Health. 2020; 5(10). DOI: 10.1136/bmjgh-2020-003648 PMC7545501 33033053

[22] Loewenson R, Accoe K, Bajpai N, Buse K, Deivanayagam TA, London L, et al. Reclaiming comprehensive public health. BMJ Glob Health. 2020; 5(9). DOI: 10.1136/bmjgh-2020-003886 PMC7520813 32978214

[23] Clay-Williams R, Rapport F, Braithwaite J. The Australian health system response to COVID-19 from a resilient health care perspective: what have we learned? Public Health Res Pract. 2020; 30(4). DOI: 10.17061/phrp3042025 33294901

[24] Dodds A. The three core elements of a better, more integrated health and economic response to COVID-19 2021 [Available from: https://blogs.lse.ac.uk/politicsandpolicy/anneliese-dodds-covid19-response/].

[25] Stein KV, Goodwin N, Miller R. From Crisis to Coordination: Challenges and Opportunities for Integrated Care posed by the COVID-19 Pandemic. Int J Integr Care. 2020; 20(3): 7. DOI: 10.5334/ijic.5595 PMC7427683 32863805

[26] Haldane V, De Foo C, Abdalla SM, Jung AS, Tan M, Wu S, et al. Health systems resilience in managing the COVID-19 pandemic: lessons from 28 countries. Nat Med. 2021; 27(6): 964–80. DOI: 10.1038/s41591-021-01381-y 34002090

[27] In focus COVID-19 Hospitalisations in NSW Reporting Period: 1 January to 19 April 2020. 2020. [Available from: https://www.health.nsw.gov.au/Infectious/covid-19/Documents/in-focus/hospitalisations.pdf].

[28] World Health Organisation COVID-19 Strategic Preparedness and Response Plan [SPRP 2021] 2021 [Available from: https://www.who.int/publications/i/item/WHO-WHE-2021.02].

[29] Australian government. Australia’s COVID-19 Vaccine and Treatment Strategy. August 2020 [available from: https://www.health.gov.au/resources/publications/australias-covid-19-vaccine-and-treatment-strategy].

[30] Julia C, Saynac Y, Le Joubioux C, Cailhol J, Lombrail P, Bouchaud O. Organising community primary care in the age of COVID-19: challenges in disadvantaged areas. Lancet Public Health; 2020. DOI: 10.1016/S2468-2667(20)30115-8 PMC7220164 32411922

