# Supplementary material for: Understanding for whom, how and why Sydney Local Health District’s Integrated Response was Effective in Addressing COVID-19: A Critical Realist Qualitative Study
Source: Int J Integr Care. 2022 Feb 11;22(1):13. doi: 10.5334/ijic.5991 (PMC8833264; doi:10.5334/ijic.5991)
Supplement: Supporting Tables. — Supporting Tables 1 and 2. [file ijic-22-1-5991-s1.pdf]

Supporting Table 1. Interview Guide.

**Exploring the agency's role and scope**	What is your role in [agency]?<br><br>What role did your agency/unit/team play in the planning and conduct of the response to the COVID 19 pandemic?	Could you describe your role and the team's role in response to the COVID-19 pandemic?
**For whom, how and why**	Upon reflection, for who did your program work for, who did it not?	Can you provide an example?
	What have been some key challenges in the implementation of the agency's response to the pandemic?	Policy level, organisational structures, provider, and consumer level
	From your perspective, what would be the key outcomes of interest your agency's role in the response to the pandemic?	How has that been evaluated?
**Exploring integration**	As you know this project, is about that big picture of how the individual agencies have played such a key part in the district's response. In the next part of the interview, we would like to better understand how these core services have been integrated.	
	What organisations have your agency worked closely with during this pandemic?	What worked well? What didn't work well? Were there other agencies you would have liked to work more closely with?
	Did your relationships with the agencies change during the pandemic?	Could you describe how this happened?
	What has been unique about the Sydney Local Health District's response to the pandemic?	
**Suggestions**	What do you think could be improved in response to the COVID-19 pandemic?	

Supporting Table 2. Nodes: SLHD Integrated Response to COVID-19.

Name	Description
**1. Complex Integrated Response**	**Includes all the roles and responsibilities of the different SLHD agencies, and a brief description of how they intersect/integrate.**
Effective quarantine	Descriptions of the agencies work in providing effective quarantine through education, RPA virtual model of care to provide high quality care to complex patients.<br><br>Subnodes: Infection control; international travellers; police hotels; rpavirtual; special health accommodation
Reaching the community	Descriptions of core agencies that are reaching the communities e.g. RPA virtual, and community wellbeing clinics, drive throughs, working across sectors, councils to deliver education, screening and testing, mobile vans, boarding houses and sleeping rough.<br><br>Subnodes: Aged care; boarding houses and sleeping rough; community; community health; community wellbeing clinics; GPs and PHN; hotline, discharge support team; outreach team; population health; pop-up clinics
Strong core infrastructure	This includes the logistics necessary to manage the response such as ICT, pathology capacity, management and communication.<br><br>Subnodes: Governance; ICT; pathology; strategic relations: tiger teams
Testing and screening	This includes the work done by the public health unit, flying squad, drive through clinics, education of the publics, and provision of results.<br><br>Subnodes: Absorb the increasing workload; drive through clinics; flying squad; pop up clinics; public health unit; clinics for staff
**2. Context**	**Description of the macro, meso and micro level contextual issues that affected the design, implementation of the different agencies' responses and integration within SLHD and with external agencies.**
Community and individual	Individual level context, communities' perspectives towards to the pandemic such as use of mask, fear, motivation to be in it together. Established respectful partnerships and relationships<br><br>Subnodes: A very human time in how people responded to COVID e.g. donations; Daily concerns e.g. food shortage; epidemiology; marginalised populations; political and community pressure; stigmatisation
Global and national	What is happening globally, at the national level that impacted on SLHD's response, across the timelines since March. [e.g. rising mortality in high income countries, fear around the ICU bed shortage, PPE stock].<br><br>Subnodes: Changing demographic of returning Australians; Closure of borders, Command and Control [Government] & SHEOC; international and COVID response; PPE equipment and consensus in terms of guidelines; recent bushfire season in NSW; second wave in Melbourne; Telehealth Medicare Schedule; vulnerable communities

Name	Description
Local	Organisational level at the SLHD level that is affecting the response. [e.g. underlying context of RPA Virtual being piloted, the integrated care plan within SLHD, disaster planning]<br><br>Subnodes: Chief executive; command control communications; community health structure; elective surgeries; established pandemic and disaster plan; establishing telehealth in 2019; existing partnerships with housing, land, DCJ; existing relationships in community; links to the ministry and to other health districts; location in relation to jurisdiction for airport and white bay; open to innovation; rpa virtual; strong existing communication pathways; strong focus on staff wellbeing; strong underlying population health management; support from the community; this is what we do
**3. Implementation barriers and facilitators**	**This includes common themes across the agencies about what were the challenges and what was done well.**
Barriers	Subnodes: Fatigue; funding; increased workload; lack of time for business as usual; lack of documentation; lack of structure or plan [beginning of pandemic]; lack of support; politics and blame; lack of time to debrief; limited capacity; overwhelming amount of information through emails; responding during a time of uncertainty and changing guidance
Facilitators	Subnodes: Decrease in demand of regular services; empowerment; Established communication channels and decision making processes; existing relationships, existing triage structure, experience – past epidemics; flexibility of staff; focus on staff wellbeing; funding; increasing capacity; IT of high quality; mobilising external partnerships; multidisciplinary team; standard operating procedure; support from CE; telehealth; workforce deployment
**4. Mechanisms of the integration**	**Includes how and why the response was integrated.**
Building on relationships	Descriptions of the relationships between individuals and agencies. [e.g. how these were strengthened and intensified]
Cautious approach	Descriptions of how the district approached the pandemic in a cautious manner [e.g. strict local discharge policy for those in the special health accommodation, resulting in the establishment of a discharge team].
Change in perception of the districts technological maturity	Descriptions of how telehealth is better recognised and valued.
Common vision	Overarching observations that we are in this together, and to help each other. This included staff from SLHD, outside, and also community. This resulted in 'talking immediately' to other departments and across hierarchy.
Coordinating care	Descriptions of how coordination was triggered, through activities such as RPA Virtual and the interagency plans

Name	Description
Empowering and engaging the community	Descriptions of how many of the activities sought to empower the community, reduce anxiety, and enable self-quarantine and management, e.g. consistent messaging, testing, quick results, information across languages.<br><br>Subnodes: Reaching CALD and marginalised populations; working with community leaders
Governance and leadership	Perspectives of governance and leadership across different levels mattered, especially the role of the chief executive within SLHD, and leadership from NSW Health.<br><br>Subnodes: Accountability for budget; across NSW health and also across sectors; need for local decision making due to uncertainties
Reorientation of care towards the community	Having activities being in the community to improve access and outcomes. This includes the work done to establish drive throughs, the community wellbeing clinics, mobile vans.
**5. Outcomes of the integrated SLHD response**	Participants' descriptions of what the outcomes are from the integration of the response.
Help Australians to reduce their own risk and the risk to their families and communities	Subnodes: Identification & Rx of vulnerable populations [D&A, expats]; reduce community anxiety
Minimise how sick these people become and how many people die	Subnodes: Deaths; increased virtual and telehealth care
Minimise the number of people [community and staff] being sick with COVID	Subnodes: Earlier detection of cases, effective containment, increased testing, opportunistic health checks, reduce hospital presentations and admissions
Reduce the burden on our health systems, to be able to provide regular care	Subnodes: Increased efficiency; increased importance and power of SLHD; keeping people out of hospital; meeting demand; reduce staff anxiety; reduced reliance on ED and hospital; reduced transmission of other infectious diseases; reduction in regular services and capacity; training opportunities
Unintended consequences	Subnodes: Increased crime, negative outcomes
Whole of health approach	Subnodes: Better communication as an organisation; connections with the councils; district and state knowledge exchange; full district level digital health strategic plan; greater appreciation and awareness of public health; greater appreciation of community health; greater appreciation of telehealth and virtual care; Increased awareness of what can be achieved; increased partnerships;

Name	Description
	increased recognition of elderly; increased understanding and relationships between staff; new skills; raised awareness of community health; research and evaluation; staff wellbeing; sustainable solutions beyond pandemic; working with the ministry
